# Validity of aerosolization detection with an air quality indicator in noncontact tonometry using corneal phantoms

**DOI:** 10.3389/fopht.2022.1021725

**Published:** 2022-10-07

**Authors:** Jonathan T. Ibinson, Atieh Yousefi, Cynthia J. Roberts, Matthew A. Reilly

**Affiliations:** ^1^ Department of Biomedical Engineering, The Ohio State University, Columbus, OH, United States; ^2^ Department of Ophthalmology and Visual Sciences, The Ohio State University Wexner Medical Center, Columbus, OH, United States

**Keywords:** aerosols, COVID-19, intraocular pressure, noncontact tonometry, corneal phantoms

## Abstract

**Précis:**

Using a controlled experimental design with corneal phantoms, this study provides evidence of the lack of validity of a static air quality indicator, previously used to characterize aerosolization during dynamic noncontact tonometry.

**Purpose:**

To evaluate the accuracy of aerosol concentrations reported by an air quality indicator (AQI) following an air puff from a noncontact tonometer using non-aerosolizing corneal phantoms.

**Methods:**

Three rubber corneal phantoms of different stiffnesses were used to represent varying intraocular pressure (IOP) values. No liquid components and therefore no aerosol-generating potential was present. Reported concentrations of particulate matter (PM) having diameter less than 2.5 and 10µm, respectively PM2.5 and PM10, were recorded using an AQI before and during an air puff generated using noncontact tonometry. The effects of covariates IOP and sensor location on changes to air quality measurements from the baseline were evaluated using analysis of variance. Monte Carlo simulations were used to determine the likelihood of observing published trends by chance. The statistical significance threshold was p<0.05.

**Results:**

No correlations were found between PM2.5 and IOP or location. Reported concentrations of PM10 depended significantly on both IOP (p=0.0241) and location (p=0.0167). Monte Carlo simulations suggest the likelihood of finding a spurious positive correlation between IOP and PM at the upper same location are 53% and 92% for PM2.5 and PM10, respectively, indicating the AQI has systematic bias resulting from non-aerosol sources.

**Conclusions:**

We were able to reproduce the published correlation between reported aerosol concentration and IOP in non-contact tonometry using dry rubber phantoms in place of living corneas with tear films. In this study, we demonstrated that published correlations linking NCT to tear film aerosolization were artifacts of the measurement technique.

## Introduction

Throughout the COVID-19 pandemic, there has been much discussion around the risk of infectious transmission during noncontact tonometry (NCT), as it was shown COVID-19 infection is possible through ocular sources (1). Protective methods have been widely recommended, including but not limited to the following: wearing of personal protective equipment (2), avoiding NCT in routine settings (2), and use disinfectant wipes of at least 70% alcohol if intraocular pressure (IOP) measurement is necessary (2). Note that damage to tonometer prisms is possible from these disinfectants (2). A recent study (3) reported the creation of aerosols from the tear film while human subjects underwent NCT. In this risk-discovery study (3), measurements were made with an air quality indicator (AQI) placed adjacent to the participant’s eyes​. Further, the study also reported a positive correlation between IOP and aerosol generation (3). A subsequent study by the same group using the same AQI further investigated the effect of intraocular pressure (IOP) on aerosol generation (4). If correct, and aerosols are being generated during NCT, then this will impact the standard of care not only in COVID-19 protection plans, but also in future incidences of airborne diseases to ensure the safety of the medical professionals. Therefore, if NCT generates risk for transmission of airborne diseases, this may change the risk profile for IOP measurement. In the current study, the validity of published findings was tested by evaluating the use of an AQI to measure aerosolization of a dynamic NCT air puff and replicating the aforementioned reports (3, 4) in a controlled setting, where any source of aerosolization is eliminated by replacing the human eyes with dry, inert corneal phantoms.

## Materials and methods

### Experimental setup

**Figure 1:**
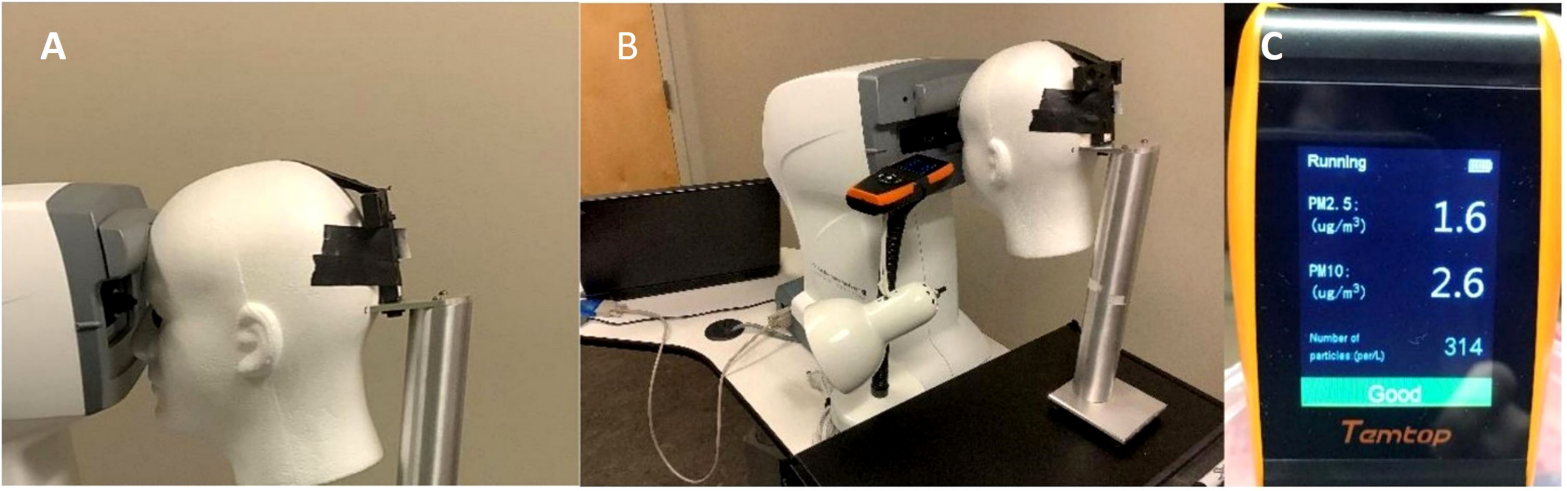
**(A)** Corneal Phantom mounted in left eye socket of Styrofoam headform. **(B)** Placement of the Styrofoam headform with the Ocular Response Analyzer and Air Quality Indicator in the Upper Same Position. **(C)** Example Output of the Air Quality Indicator following a Measurement on the Corneal Phantom.

Three rubber corneal phantoms of different stiffnesses were used throughout the experiment to represent IOP values of 6, 13, and 43 mmHg with these three values being assigned to the phantoms by Reichert Technologies (Depew, NY, USA), calibrated by the amount of air puff load required to applanate their surfaces using an Ocular Response Analyzer G3 (ORA) (Depew, NY, USA). Phantoms were manufactured with a proprietary molding process of various material properties and thicknesses to mimic Goldmann Applanation Tonometry values measured in human eyes and are currently used in-house by Reichert to calibrate noncontact tonometers. Phantoms were stored in a closed container to protect them from contamination and visually inspected prior to use. The maximum air puff magnitude produced by the ORA is a function of the patient’s IOP and corneal stiffness with greater IOP and stiffness resulting in greater maximum air puff magnitude (5). Thus, the air puff is customized such that the individual with higher IOP would receive a greater air puff magnitude. The phantoms do not have any liquid component and therefore do not have an aerosol-generating potential. Each phantom was securely mounted inside the eye socket positions of a Styrofoam head, seen in **Figure 1A**. Phantom locations were recorded relative to right eye vs left eye position. The Styrofoam head was placed in front of an ORA and aligned in the same manner as a patient, seen in **Figure 1B**. An AQI (LKC 1000S+; Milpitas, CA, USA) was placed adjacent to the foam head and recorded air quality measurements consisting of concentrations of particulate matter (PM) less than 2.5 and 10 µm, respectively, denoted as PM2.5 and PM10. The AQI was placed in four locations around the phantom: eye-level and slightly below eye-level on the left and right side. Depending on indicator position with respect to the phantom being tested at that time, the locations were defined as upper or lower regarding the height and same or opposite regarding the side.

The ORA generated an air puff on one of the eye phantom locations, and the AQI measurements were taken during the noncontact tonometry. Baseline AQI measurements were recorded before each test. In order to avoid introduction of new aerosols during the data acquisition process, several precautionary measures were taken: a one-minute delay was allotted between tests to allow the device to recalibrate; data collection of each phantom was completed in its entirety before measurements for a new phantom was started; the tests were conducted in a temperature and humidity-controlled environment in a research room not used for patient care, with the door closed; the head was placed away from the door; the researchers wore surgical masks for the entirety of the testing; and the researchers did not speak during the tests to keep airflow in the room as low as possible. Each location of each phantom had the tests repeated four times. Because the baseline measurement was taken before each test, a total of eight measurements were taken per location per phantom. At four locations for three phantoms, this gave a total of 96 measurements.

### Statistical analysis

Analysis of variance (ANOVA) was used to assess the effect of covariates IOP and location on changes to AQI measurements from the baseline, ΔPM2.5 and ΔPM10. A Monte Carlo simulation with 10,000 replicates at each phantom/measurement location combination was undertaken to determine the likelihood of observing published trends by chance. Inputs for these simulations were experimentally determined means and standard deviations for each phantom, and measurement location. Outputs for these simulations were simulated AQI estimates of PM2.5 and PM10. The statistical significance threshold was p < 0.05.

## Results

No correlations were found between PM2.5 and IOP (p = 0.3943) or location (p = 0.3049). Significant correlations were found between PM10 and IOP (p = 0.0241) and location (p = 0.0167). Changes in PM values from baseline for all locations are given in **Table 1**.

**Table 1:** Change in Particulate Matter (PM) Values by Intraocular Pressure (IOP) and Location specific phantom.

PM2.5	Change in Particulate Matter (µg/m^3^):		
Location:	6 mmHg IOP Phantom	13 mmHg IOP Phantom	43 mmHg IOP Phantom
Upper Same	0.0 ± 0.4	-0.1 ± 0.1	0.0 ± 0.3
Lower Same	0.1 ± 0.4	0.2 ± 0.3	0.1 ± 0.2
Upper Opposite	0.0 ± 0.2	-0.3 ± 0.3	0.1 ± 0.3
Lower Opposite	0.1 ± 0.2	-0.1 ± 0.2	0.1 ± 0.2
**PM10**	**Change in Particulate Matter (µg/m^3^):**		
**Location:**	**6 mmHg IOP Phantom**	**13 mmHg IOP Phantom**	**43 mmHg IOP Phantom**
Upper Same	0.2 ± 0.7	0.1 ± 0.1	0.4 ± 0.4
Lower Same	0.2 ± 0.6	0.2 ± 0.4	0.5 ± 0.3
Upper Opposite	-0.2 ± 0.4	-0.7 ± .3	0.2 ± 0.5
Lower Opposite	0.1 ± 0.3	0.1 ± 0.3	0.1 ± 0.2

Each value is the mean ± standard deviation of four tests. PM2.5 is particulate matter of size 2.5µm and PM10 is particulate matter of size 10µm. The intraocular pressure is the equivalent pressure assigned to each rubber phantom.

The Monte Carlo simulation using these results suggest that the likelihood of finding a spurious positive correlation between IOP and AQI measurements at the upper same location are 53% and 92% for PM2.5 and PM10, respectively.

## Discussion

The current experiment was designed to replicate the core components of existing literature that uses AQI in an aerosol context, specifically in studies by Tang et al (3, 4). All three studies used the same model and brand of AQI, limited outside airflow as much as possible, measured multiple locations around the AQI, and tested different IOP ranges. The main difference was the samples used, which saw the original patient eyes (3, 4) replaced with rubber phantoms in the current study.

Despite the use of corneal phantoms, which lack a tear film or other possible source of aerosols, a statistically significant correlation was found between reported PM10 and covariates IOP and location. The phantoms were shown to adequately mimic the IOP values reported by the manufacturer by the IOPcc measurements from the ORA, shown in **Table 2**. As previously described, the ORA produces a higher magnitude air puff with greater IOP, so it is likely this contributed to the spurious result using the AQI. In addition, the serious limitations of the AQI for the purpose of measuring short timescale aerosols were detailed in a Letter to the Editor, including that the reported values in the Tang study are far *below* the nominal accuracy of the AQI according to the user manual, calling these previously reported values into question (6). Further, within the Monte Carlo simulation, the upper same location was selected, given the proximity of this location to the air puff impact location and the likelihood that the highest density would be found. Monte Carlo simulation showed more than half (53%) of PM2.5 replicates and a majority (92%) of PM10 replicates displayed a significant correlation, even though no aerosol source was present. These results suggest the published IOP-PM correlations were not valid and therefore cannot be used to conclude that aerosols were generated (3, 4). The NCT used in the published studies produced different air pressure magnitudes, one for lower IOP and one for higher IOP values (6). Given that higher-pressure generates more turbulent flow, this difference in air pressure magnitude would have been present between the various IOP’s. There is the strong possibility that the false positive correlation was due to turbulent flow from the air puff rather than aerosol generation from the corneal surface. Since the AQI relies on a light scattering sensor, turbulence-induced variation in the refractive index of the air itself must be considered a confounding factor (7).

**Table 2:** Experimental vs. manufacturer intraocular pressure (IOP) values.

Phantom	Average IOPcc (mmHg)	Manufacturer Listed IOP (mmHg)
Blue	5.9 ± 0.4	6
White	11.5 ± 0.4	13
Red	43.0 ± 0.6	43

Each average IOPcc value is the mean ± standard deviation of 36 tests.

Other technical issues exist in using the AQI to quantify aerosolization. Per the AQI user manual, the accuracy of the particulate matter measurements is ±10 µg/m^3^ and ±15 µg/m^3^ for PM2.5 and PM10, respectively (7). However, our data were consistently around 1–3 µg/m^3^ for both PM2.5 and PM10, shown in **Figure 1C**. Further, the NCT air puff duration is approximately 30 milliseconds (8). ​However, per the AQI manual, the AQI only updates every 3 seconds (7), not providing enough temporal resolution to capture the effect of the air puff impact on the cornea.

Limitations included that we did not measure temperature or humidity in the room where the experiment was conducted. However, all experiments took place in a temperature and humidity-controlled research room not used for patient care with the door closed, in order to minimize environmental variation between each experimental configuration. Also, we recognize that in this study, we are unable to conclude if aerosols are generated or not, only that the AQI is unreliable to detect them.

Our assessment is that the published reports with patients displayed a false-positive result due to the mismatch of timing between the 30ms duration of the air puff which is 1/100 of the 3s measurement window of the AQI. This statistical error means that even though a statistically significant correlation was found in the data analysis, it is not a true difference and is within the 5% error associated with a significance threshold of p < 0.05. The AQI is not capable of quantifying aerosolization with an air puff tonometer due to the multiple factors described. The current study demonstrates significant differences using corneal phantoms without aerosol-generating potential, highlighting that a quasi-static (meaning that its sampling interval is very long relative to the event being monitored: 3 s relative to ~30 ms) AQI is not appropriate to measure a high-speed dynamic process. More appropriate technology would include advanced high-speed cameras much faster than the air puff duration, paired with imaging software (9) or shadowgraphy (10) which show consistent findings of no droplets being generated in natural settings unless external drops are added that would increase the tear load (9, 10).

In conclusion, this study comprises the appropriate negative control for the prior studies of Tang et al. (3, 4) demonstrating that their conclusions were based on a Type 1 error (false positive) rather than a true increase in aerosol concentration. This experiment demonstrated that correlations linking NCT to tear film aerosolization were artifacts of the measurement technique.

## Data availability statement

The raw data supporting the conclusions of this article will be made available by the authors, without undue reservation.

## Author contributions

JI, AY, CR, and MR contributed to the manuscript. All contributed to the study design, analysis and interpretation. JI and AY collected the data. All authors contributed to the article and approved the submitted version.

## Funding

The research was partially supported by NIH/NEI R01 EYE027399.

## Acknowledgments

We would like to thank Reichert Technologies for supplying the corneal phantoms​.

## Conflict of interest

CR is a consultant for Oculus Optikgeräte GmbH (Wetzlar, Germany) and Ziemer Ophthalmic Systems AG (Port, Switzerland). She has received Honoraria from Heidelberg Engineering, Inc, and Reichert Technologies.

The remaining authors declare that the research was conducted in the absence of any commercial or financial relationships that could be construed as a potential conflict of interest.

## Publisher’s note

All claims expressed in this article are solely those of the authors and do not necessarily represent those of their affiliated organizations, or those of the publisher, the editors and the reviewers. Any product that may be evaluated in this article, or claim that may be made by its manufacturer, is not guaranteed or endorsed by the publisher.
